# Tear lipid layer deficiency associated with incomplete blinking: A case report

**DOI:** 10.1186/1471-2415-13-34

**Published:** 2013-07-16

**Authors:** Motoko Kawashima, Kazuo Tsubota

**Affiliations:** 1 MGD Clinic, Keio University School of Medicine, Tokyo, Japan; 2 Department of Ophthalmology, Keio University School of Medicine, 35 Shinanomachi, Shinjuku, Tokyo 160-8582, Japan; 3 Minamiaoyama Eye Clinic, Tokyo, Japan

**Keywords:** Lipid layer, Blink, Meibomian gland

## Abstract

**Background:**

Meibomian gland obstruction induces hyposecretion of tear film lipids, which results in lipid layer deficiency and evaporative dry eye. Unfortunately, the importance of blinking in meibomian gland dysfunction has been largely overlooked, and it is not known whether incomplete blinking causes tear lipid deficiency, even in the unobstructed meibomian glands.

**Case presentation:**

A 38-year-old woman suffering from foreign body sensations in her eyes was examined. The cornea was clear and tear secretion was normal. Lid margin abnormalities were not observed and the meibum was clear. However, the lipid layer was very thin, and the patient was given a diagnosis of incomplete blinking. The patient was made aware of her condition and asked to blink consciously and completely. After that, an immediate increase in lipid flow was observed.

**Conclusion:**

Tear lipid layer deficiency can occur with incomplete blinking, even though meibomian gland structures are intact. This case highlights the importance of complete blinking.

## Background

Dry eye disease (DED) is a common, yet complex condition that is most often caused by meibomian gland dysfunction (MGD) [1]. Meibomian gland obstruction and gland drop out contribute hyposecretion of tear film lipids, which results in lipid layer deficiency and evaporative DED [1,2]. Recently developed noncontact meibography allows physicians to clearly and noninvasively observe meibomian gland morphology [3]. Subjective ocular symptoms, lid margin abnormalities by slit lamp examination, diagnostic meibomian gland expression and meibomian gland structure observations by meibography are necessary for diagnosing obstructive MGD [3]. Unfortunately, the importance of blinking in MGD has been largely overlooked, and little has been reported on tear lipid deficiency caused by incomplete blinking even in the unobstructed meibomian glands.

## Case presentation

**Figure 1:**
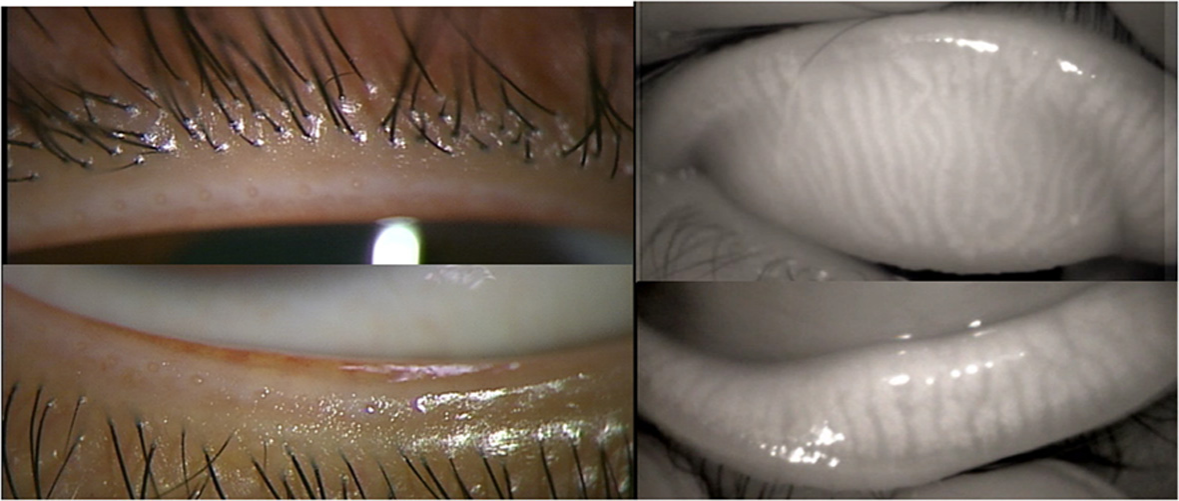
**Normal upper and lower lid margin and meibography findings.** Normal meibomian gland structures are visible.

A 38-year-old woman with a 15-year history of disposable contact lens (CL) use presented to our clinic, complaining of a foreign body sensation in her eyes. She also had > 8 hours/day of visual display terminal (VDT) exposure. Her symptom score on the Standard Patient Evaluation for Eye Dryness questionnaire (score range 0–28; for those with the score of ≧6, further DED examinations are recommended.) was 8. On slit lamp examination performed 4 hours after CL removal, the cornea was clear and no fluorescein staining was observed. Tear film break-up time was 4 seconds in the right eye and tear secretion volume measured by the Schirmer I test was 25 mm. Lid margin abnormalities, such as irregular lid margin, vascular engorgement, plugged meibomian gland orifices, and mucocutaneous junction displacement, were not observed. Clear meibum was easily expressed and noncontact meibography showed no loss of meibomian glands in either the upper or lower lids (Figure 1). Measurements and observations in the left eye were similar to those of the right eye. Neither eye met the diagnostic criteria for DED or MGD.

The LipiView Ocular Surface Interferometer® (TearScience, Inc., Morrisville, North Carolina) is a non-invasive instrument that captures live digital images of the tear film, measures its lipid content, and assesses blink dynamics. The LipiView evaluates lipid layer thickness through an Interference Color Unit score (normal average ICU score is ≥75). Surprisingly, even without CL use, this patient’s average Interference Color Unit score was 24, indicative of a very thin lipid layer.

**Figure 2:**
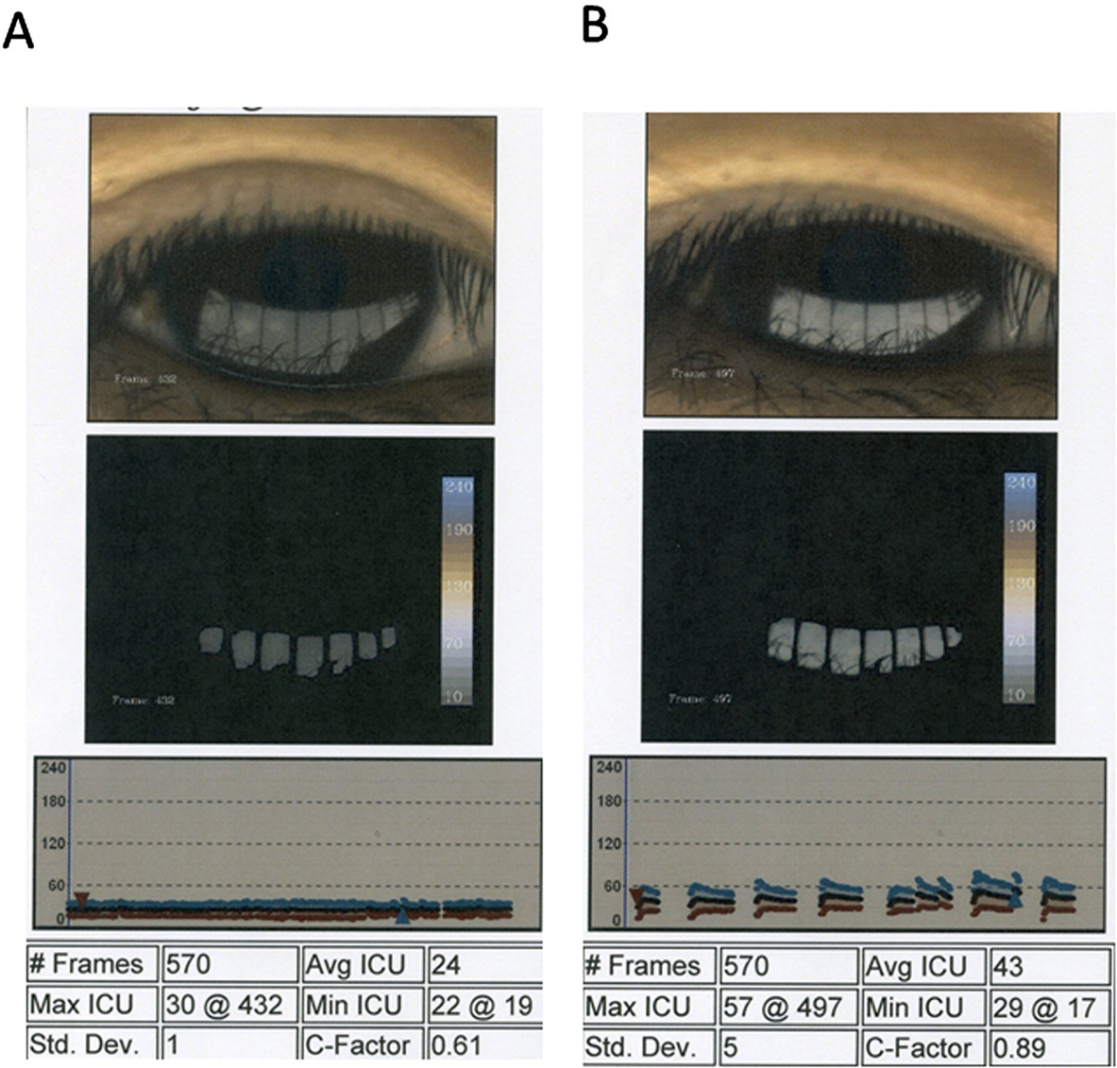
**LipiViewresults before (A) and after (B) complete blinking from a 38-year old woman, complaining of foreign body sensation.** Average Interference Color Unit (ICU) score was 24 before blinking and 43 immediately after blinking (normal average ICU score is ≥75).

The LipiView examination also revealed that the patient’s incomplete blink rate was abnormally high (Figure 2A). The patient was made aware of this anomaly and its significance and was asked to blink consciously and completely. LipiView examination was immediately performed again and a significant increase in lipid flow was observed (Figure 2B).

### Discussion

This case suggests that tear lipid layer deficiency can occur with incomplete blinking, even though meibomian gland structures are intact. We agree with previous reports that suggest that blinking efficiency affects the ocular surface health [4,5]. An incomplete blink leads to inadequate lipid distribution (thin lipid layer) as well as consequent exposure over the inferior ocular surface, which may increase evaporation. In agreement with Korb et al., [6] who reported that forceful blinking leads to significant increases in lipid layer thickness, this case demonstrates that conscious and complete blinking can also improve meibomian gland lipid flow. It also suggests that proper blinking is important in lipid layer maintenance through augmentation of meibomian gland lipid expression and lipid spreading across the tear film. Further, McMonnies CW suggested that lubricant eye drop instillation combined with blink efficiency exercises may increase the therapeutic benefit to ocular surface epithelium with the potential to improve tear distribution so that DED symptoms are alleviated and/or prevented [7].

During work at a VDT, both the blink rate and the number of complete blinks are decreased [8]. The recent marked increase in VDT exposure has also resulted in a marked increase in the number of DED patients. Furthermore, long-term CL use weakens the Muller’s muscle, which can lead to blink deficiency [9]. Therefore, more attention should be more attention should be given to blinking status, even in patients with no clinical findings for MGD (e.g., meibomian gland dropout, meibomian orifice obstructions). Although MGD mostly affects older people, lipid deficiency can occur in younger patients. In fact, lipid deficiency with no meibomian gland obstruction is estimated to be widespread in younger patients, especially those using CL and having a high VDT exposure [10,11]. In addition to current treatments, improving the blink quality and quantity may be useful in treating DED.

## Conclusions

Tear lipid layer deficiency can occur with incomplete blinking, even though meibomian gland structures are intact. This case highlights the importance of complete blinking.

## Consent

Written informed consent was obtained from the patient for publication of this case report and any accompanying images. A copy of the written consent is available for review by the Editor of this journal.

## Abbreviations

VDT: Visual display terminal; MGD: Meibomian gland dysfunction; DED: Dry eye disease; CL: Contact lens; ICU: Interference color unit.

## Competing interests

The authors have declared that no competing interests exist.

## Authors’ contributions

MK conceived of the study, performed the examination and drafted the manuscript. KT helped to draft the manuscript. Both authors read and approved the final manuscript.

## Pre-publication history

The pre-publication history for this paper can be accessed here:

http://www.biomedcentral.com/1471-2415/13/34/prepub
